# Functional Significance of Vitamin D Receptor *FokI* Polymorphism in Human Breast Cancer Cells

**DOI:** 10.1371/journal.pone.0016024

**Published:** 2011-01-24

**Authors:** Fatouma Alimirah, Xinjian Peng, Genoveva Murillo, Rajendra G. Mehta

**Affiliations:** 1 Division of Cancer Biology, IIT Research Institute, Illinois Institute of Technology, Chicago, Illinois, United States of America; 2 Department of Biological Chemical and Physical Sciences, Illinois Institute of Technology, Chicago, Illinois, United States of America; Vanderbilt University Medical Center, United States

## Abstract

**Background:**

The *FokI* vitamin D receptor (VDR) polymorphism results in different translation initiation sites on VDR. In the VDRff variant, initiation of translation occurs at the first ATG site, giving rise to a full length VDR protein of 427 amino acids. Conversely, in the VDRFF variant, translation begins at the second ATG site, resulting in a truncated protein with three less amino acids. Epidemiological studies have paradoxically implicated this polymorphism with increased breast cancer risk. 1α,25 (OH)_2_D_3_, the active metabolite of vitamin D, is known to inhibit cell proliferation, induce apoptosis and potentiate differentiation in human breast cancer cells. It is well documented that 1α,25 (OH)_2_D_3_ downregulates estrogen receptor α expression and inhibits estrogen mediated signaling in these cells. The functional significance of the VDR *FokI* polymorphism in vitamin D action is undefined.

**Methods/Findings:**

To elucidate the functional role of *FokI* polymorphism in breast cancer, MCF-7-Vector, MCF-7-VDRff and MCF-7-VDRFF stable cell lines were established from parental MCF-7 cells as single-cell clones. In response to 1α,25 (OH)_2_D_3_ treatments, cell growth was inhibited by 60% in VDRFF cells compared to 28% in VDRff cells. The induction of the vitamin D target gene *CYP24A1* mRNA was 1.8 fold higher in VDRFF cells than in VDRff cells. Estrogen receptor-α protein expression was downregulated by 62% in VDRFF cells compared to 25% in VDRff cells. VDR protein stability was greater in MCF-7-VDRFF cells in the presence of cycloheximide. PCR array analyses of VDRff and VDRFF cells revealed increased basal expression levels of pro-inflammatory genes *Cyclooxygenase-2, Interleukin-8 and Chemokine (C-C Motif) Ligand 2* in MCF-7-VDRff cells by 14, 52.7 and 5 fold, respectively.

**Conclusions/Significance:**

These results suggest that a VDRff genotype may play a role in amplifying aggressive breast cancer, paving the way for understanding why some breast cancer cells respond inefficiently to vitamin D treatment.

## Introduction

The onset and progression of breast cancer is multifactorial and not fully defined. It is well established that 1α,25(OH)_2_D_3_ (1,25D_3_), the active metabolite of vitamin D, plays a pivotal role in negatively affecting breast cancer cells by inhibiting cell proliferation, curtailing invasiveness, inducing apoptosis and potentiating differentiation [1]. Furthermore, lower circulating levels of vitamin D in women have been positively linked with enhanced breast cancer risk and disease mortality [2], [3].

Vitamin D action is mediated by the nuclear receptor and transcription factor Vitamin D receptor (VDR). Upon binding to 1,25D_3_, VDR heterodimerizes with RXR, another nuclear receptor, and together they bind to specific vitamin D response elements (VDREs) in promoter regions of vitamin D target genes, executing transcriptional effects [1]. Alternatively, in a vitamin D independent manner, VDR itself has also been shown to dimerize with RXR and regulate specific target genes [4]. Importantly, experimental studies on mammary tumors derived from mice lacking VDR have shown it necessary for vitamin D action as 1,25D_3_ failed to inhibit cell proliferation and apoptosis in these cells [5].

Consistent with its essential role in vitamin D mediated effects on breast cancer, several polymorphisms in the VDR gene have been identified and their possible significance in breast cancer has been inconclusively assessed in epidemiological investigations across multi-ethnic groups [6], [7]. One such polymorphism is the *FokI* polymorphism restriction site located on exon 2 in the 5′ coding region of the gene [6]. This polymorphism results in different translation initiation sites on VDR. A thymine (T) to a cytosine (C) conversion in the first translation initiation codon ATG (methionine) generates long and short variants of VDR. In the VDRff variant initiation of translation occurs at the first ATG site, giving rise to a full length VDR protein comprised of 427 amino acids. Conversely, in the VDRFF variant translation begins at the second ATG site instead of the first, resulting in a truncated protein with three less amino acids. This is the only known VDR polymorphism resulting in two different VDR protein products [6].

The *FokI* polymorphism, either singly or in combination with other VDR polymorphisms, has been extensively investigated in breast cancer risk assessment studies [7]–[13]. For example, Guy *et al* reported that the *FokI FF* allele together with other VDR polymorphisms, amplified breast cancer risk in a Caucasian population in the United Kingdom [8]. On the other hand, two other studies found that women with the *ff* genotype were more susceptible to breast cancer than those with the *FF* genotype [9], [10], while another study did not observe any correlation between the *FokI* polymorphism and increased breast cancer risk in postmenopausal women [11]. These conflicting conclusions are often derived due to small sample sizes, compounding variables and selection biases in patient populations for each study. However, more recently, two reports with meta-analyses of multiple studies with large sample sizes provide evidence for a positive association between the *FokI ff* genotype and an augmented predisposition to the disease [12], [13]. However, these reports do not provide any conclusive evidence linking either the VDRff or VDRFF variant to breast cancer risk or responsiveness to vitamin D. Therefore, it is necessary to evaluate functional differences between polymorphic alleles experimentally in breast cancer cells.

In the present study, we established three cell lines from single cell clones: Vector control and cells stably overexpressing VDRff and VDRFF variants in parental MCF-7 cells and determined their functional significance in breast cancer. This is the first report documenting a differential response to 1,25D_3_ in relation to cell proliferation, transactivation of vitamin D target gene, *CYP24A1* and modulation of estrogen receptor signaling among the two VDR alleles in breast cancer cells. We also report differential basal expression of pro-inflammatory genes in VDRff and VDRFF overexpressing breast cancer cells, which may be responsible for the amplified genetic susceptibility to an aggressive form of the disease.

## Materials and Methods

### Cell culture and reagents

The MCF-7(ER+, PR+,VDR+) and the MDA-MB231 (ER-, PR-, VDR+) human breast cancer cell lines were purchased from the American Type Culture Collection (Manassas, VA). The cell lines were maintained in MEM medium (Invitrogen Life Technologies, Carlsbad, CA) supplemented with 10% Fetal Bovine Serum, 0.01% non-essential amino acids and antibiotics. 1,25D_3_ was purchased from Cayman Biochemicals (Ann Arbor, MI); whereas 17β-estradiol (E2) and tamoxifen were obtained from Sigma-Aldrich Corp., (St. Louis MO). Cycloheximide was purchased from A.G. Scientific INC, (San Diego, CA).

### Single clonal cell establishment

Parental MCF-7 cells were subjected to serial dilutions in 96 well plates to obtain a single cell-colony per well. Expanded single colonies were transferred to a 24 well plate and subsequent clones were further isolated as single cells using clonal cylinders. These colonies were then transferred to 12 well plates and subsequently subjected to immunoblotting for basal VDR protein expression. The clone expressing the lowest basal VDR protein was selected for stable transfection.

### Stable cell line generation and treatment

**Figure 1:**
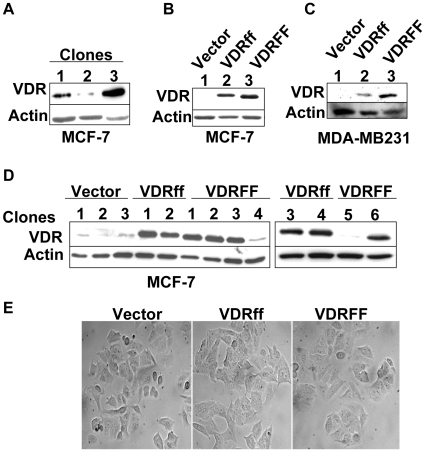
Generation of stably transfected MCF-7 cell lines with Vector, VDRff and VDRFF genotypes. (A) Single clones were isolated from parental MCF-7 cell lines by serial dilutions in 96 well plates and clonal proteins were analyzed for basal VDR levels by immunoblotting as described in Materials and Methods. (B) MCF-7 parental clone expressing the lowest VDR protein basal level (clone 2 from Fig. 1A) was selected for stable transfection with the indicated plasmids and pooled protein samples were analyzed for VDR protein expression. (C and D) Single clones were selected from stably transfected MCF-7 cells (C) and pooled MDA-MB231 clones (D) were processed for VDR protein expression by immunoblotting. (E) MCF-7 Vector, MCF-7 VDRff and VDRFF single clones were visualized at the same passage by phase contrast microscopy at 40× magnification.

The above mentioned clone was stably transfected in 60 mm plates with 5 µg of pcDNA3.1 Vector control, VDRff and VDRFF expression constructs using Lipofectamine 2000 (Invitrogen) per the manufacturer's instructions. The full length pcDNA3.1 human VDRff was generously provided by Dr. Xiao-Kun Zhang (Burnham Research Institute, La Jolla, CA). The *FF* allele construct was generated from the VDRff plasmid by site directed mutagenesis by utilizing the QuickChange site directed mutagenesis kit (Stratagene, La Jolla, CA). Two complimentary oligonucleotides of 30 nucleotides each were used. These nucleotides spanned the ATG initiation codon and differed from the template sequence by an ACG instead of an ATG. The entire VDR coding sequence of both VDRff and VDRFF was verified by DNA sequencing. Twenty four hours post-transfection, the cells were split into 100 mm plates and selected with 800 µg/ml G418 (RPI, Palos Heights, IL) for one month. Forty eight G418 resistant single clones were isolated with clonal cylinders and cultured in 24 well plates and thirteen of these independent clones as well as pooled clones were successfully established as cell lines designated as MCF-7-Vector, MCF-7-VDRff and MCF-7-VDRFF. The VDR protein expression for each of these cell lines was determined by western blot analysis. The Vector, VDRff and VDRFF clones were randomly selected and designated as clones 1, 2 and 3 as outlined in Figure 1C. The numbers above the figure correspond to the clone number. To circumvent artifactual effects, where indicated, two to three individual clones were analyzed. All clones were maintained in medium containing 200 µg/ml G418 and all experiments were conducted between passages 4 and 15. The MDA-MB231 cells overexpressing VDRff and VDRFF were transfected using a similar approach and pooled colonies were utilized. All cells were visualized at 40× magnification by phase contrast microscopy (Olympus DP70).

### Western blot analysis and co-immunoprecipitation

Total cell lysates were prepared and subjected to western blot analysis as previously described (14). VDR rat monoclonal (Clone 9A7y.E10.E4) antibody was purchased from Neomarkers (Freemont, CA). Antibodies specific for ERα (sc-8005), RXRα (sc-553), control anti-IgG (sc-2027) and β-Actin (sc-1616) as well as all secondary antibodies were purchased from Santa Cruz Biotechnology (Santa Cruz, CA). Immunoprecipitation was performed as previously described [14].

### Cell proliferation analysis

Cell proliferation was assessed by the crystal violet assay and cell counting as previously described [15]. In experiments assessing estrogen receptor mediated signaling, cells were incubated with phenol free MEM medium supplemented with 5% charcoal stripped serum, 0.01% MEM-non-essential amino acids, 0.01% L-glutamine and antibiotics (Invitrogen). The cells were treated with either 1,25D_3_ (100 nM), 17β-estradiol (10 nM) or tamoxifen (1 µM) for 4 and 7 days and subjected to the crystal violet assay and Absorbance was ascertained at 570 nM as previously described [15]. The cell counting was carried out using the Z1 Coulter Particle Counter (Beckman Coulter, Fullerton, CA).

### Reporter assays

The *CYP-24* promoter-luciferase reporter plasmid was generated by isolating genomic DNA via PCR. Approximately 400 base pairs of the 5′ flanking region (−296/+109 relative to the transcription start site) of CYP24 was used. The genomic DNA was extracted from MDA-MB435 cells using Advantage2 PCR kit (Clonetech). The *CYP24* was cloned to the kpn/BgI II sites of the promoterless pGL3 basic vector (Promega, Madison, WI). MDA-MB231 cells were transiently transfected with 0.3 µg *CYP24-luc*, 0.3 µg either of VDRff or VDRFF plasmids and 10 ng *phRL-TK* internal control per well in 12 well plates using Lipofectamine 2000 (Invitrogen) per the manufacturer's instructions. Twenty four hours post-transfection, cells were incubated in the presence or absence of 100 nM 1,25D_3_ for twenty four hours and subsequently assessed for the firefly and *Renilla* luciferase activities using the Dual- Luciferase Reproter Assay Kit (Promega, Madison, WI). The firefly luciferase activity was normalized to *Renilla* luciferase activity.

### qRT-PCR analysis and PCR Array

Following the experimental treatments, total RNA was isolated from the cells using TRIzol reagent (Invitrogen) as suggested by the supplier. cDNA synthesis and qRT-PCR analysis was conducted as previously described and all samples were normalized to Actin control [16]. The Signal Transduction Pathway Finder PCR Array of 84 genes (SA, Biosciences, Frederick, MD) was performed according to the manufacturer's instructions. For the PCR array, cells were processed as above and total RNA was digested with DNase I to eliminate chromosomal DNA contamination (Qiagen, Valencia, CA) and purified using the Qiagen RNeasy Mini Kit per the manufacturer's instructions. RNA integrity and quality was determined prior to gene expression analysis. The manufacturer's web-based software package was utilized to calculate fold changes. Genes with greater than 2 fold regulation were confirmed by qRT-PCR. The primers were designed based on the gene identification number outlined in the PCR array.

### Statistical analysis

Statistical significance was analyzed by one-way ANOVA using the GraphPad Software (San Diego, CA). Tukey's test for multiple comparisons was used for all post-analyses. Differences between means were considered significant when *P<0.05 or better. The data are presented as mean values ±SD.

## Results

### Sequential establishment of Vector, VDRff and VDRFF constitutively expressing cell lines

The parental MCF-7 cell line is comprised of a heterogeneous population [17] endogenously expressing the VDRFF variant [8]. To decrease endogenous VDR protein background, a cell line expressing the lowest basal VDR protein expression was established from parental MCF-7 cells. Single clones were isolated as described in Materials and Methods. Figure 1A illustrates various basal VDR protein levels. Clone 2, which expressed the lowest basal VDR protein, was selected to generate Vector control or cell lines overexpressing VDRff and VDRFF variants. Figures 1B &1C depict VDRff and VDRFF pooled colonies (B) as well as individual clones (C). The expression of VDRff and VDRFF in MDA-MB231 pooled clones is illustrated in Figure 1D. As shown in the figures, in cells overexpressing VDRFF, the VDR protein was approximately 0.4 kDa shorter than the cells overexpressing VDRff. This is consistent with the size of the VDR variant, which is shorter by three amino acids. As shown in Figure 1E all the three MCF-7 cell lines were morphologically similar regardless of the VDR genotype, suggesting that the VDR *FokI* polymorphism does not affect cellular phenotypes.

### Differential inhibition of cell growth in VDRff and VDRFF cells in response to 1,25D_3_

**Figure 2:**
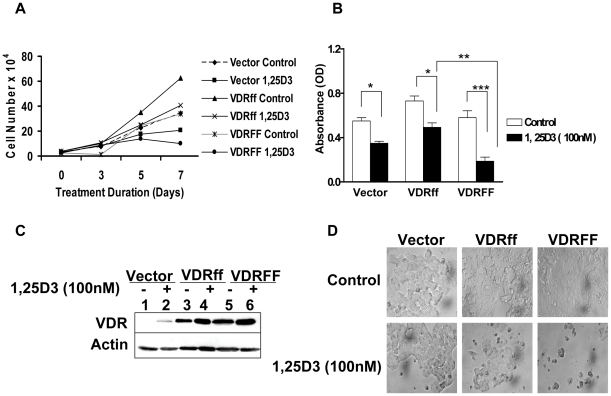
Differential inhibition of cell growth in VDRff and VDRFF cells in response to 1,25D_3_. (A) The cells were grown in triplicates and treated with 100 nM of 1,25D_3_ for 0-7 days and subjected to cell counting on days 0, 3, 5 and 7. (B) The cells were treated with 100 nM of 1,25D_3_ for 7 days and cell proliferation was determined by the crystal violet assay. The data represent analysis of three independent clones with duplicate analyses of each clone. Bars, mean ±SD; *P<0.05, **P<0.01, ***P<0.001 (one-way ANOVA test). (C) The VDR expression was determined by western blot analysis in the cells after incubating cells with 1,25D_3_ for 7 days. (D) Morphology of the cells 7 days post 1,25D_3_ treatment at 40× magnification.

To compare the effect of vitamin D treatment on the proliferation of MCF-7 cells expressing Vector, VDRff and VDRFF, the cells were incubated in the absence or presence of 1,25D_3_ and counted on days 0, 3, 5 and 7 of treatment. As shown in Figure 2A, a maximum growth inhibition of 60% (P<0.01) in response to 1,25D_3_ on day 7 was observed in VDRFF cells compared to 23% (P<0.05) and 28% (P<0.05) growth inhibition for the Vector and VDRff cells respectively. To confirm that the effects observed for these cell lines were consistent with each selected VDR genotype, two additional single clones along with the initially characterized clone, were treated with 1,25D_3_ for 7 days and subjected to the crystal violet assay. Similar growth inhibition patterns were observed for all three independent clones. The results shown in Fig. 2B are representative of three individual clones. The differences in growth inhibition between VDRFF and VDRff were statistically significant (P<0.01). It is important to note that although VDRff and Vector cells were less responsive to 1,25D_3_, their cell growth was significantly inhibited (Vector control vs. treatment, P<0.01, VDRff control vs. treatment; P<0.05). Furthermore, to discern the effect of 1,25D_3_ on VDR protein expression during maximum growth inhibition, the three cell lines were treated with 100 nM 1,25D_3_ for 7 days and subjected to western blotting. As illustrated in Figure 2C, VDR protein levels were constantly upregulated in all the cell lines. VDR induction in response to 1,25D_3_ was highest in VDRFF cells followed by VDRff cells with the Vector containing the lowest level of induction as expected. Thus, these observations indicate that VDR plays an essential role in 1,25D_3_ mediated growth inhibition. Collectively, these results imply that VDRFF cells are more sensitive to vitamin D treatment compared to VDRff cells and provide a platform for further examining the functional significance of the *FokI* VDR polymorphism in human breast cancer.

### 1,25D_3_ differentially regulates *CYP24A1* and has no effect on *CYP27B1* transcription in *FokI-*VDR polymorphic cells

**Figure 3:**
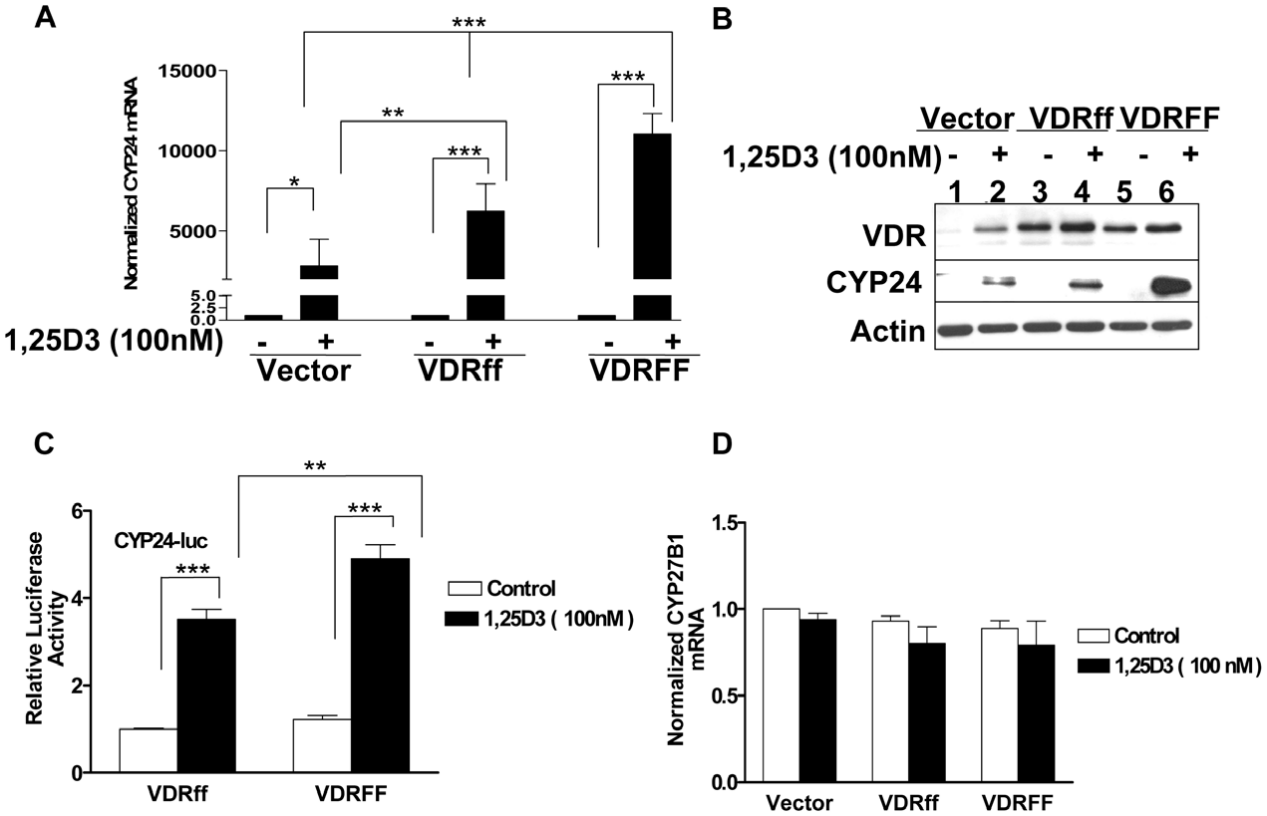
Effects of 1,25D_3_ on the expression of CYP24 and *CYP27B1* in response to 1,25D_3_ in MCF7 cells expressing VDR polymorphism. (A) The cells expressing Vector, VDRff or VDRFF were treated with 100 nM of 1,25D_3_ for 24 h; total RNA was subjected to qRT-PCR using primers specific for the *CYP24A1* gene. The data represent analyses of three independent clones. Each experimental control was set up as 1 and each experimental treatment was normalized to its control. Bars, mean ±SD; *P<0.05, **P<0.01, ***P<0.001 (one-way ANOVA test). (B) Total cell lysates from the same cells were processed for the indicated proteins. The data represent analyses of two independent clones (C) MDA-MB231 cells were plated in 12 well plates in duplicates and transiently transfected with 0.3 µg of human *CYP24* reporter plasmid, 0.3μg each of VDRff and VDRFF plasmids together with 10 ng of phRL-TK internal control using Lipofectamine 2000 transfection reagent (Invitrogen). 24 h post-transfection, cells were treated with 100 nM of 1,25D_3_ for 24 h. Subsequently, firefly and *Renilla luciferase* were determined. The normalized luciferase activity is shown. Bars, mean ±SD; **P<0.011, ***P<0.001 (one-way ANOVA test). (D) The indicated cells were treated with 100 nM of 1,25D_3_ for 24 h and total RNA was subjected to qRT-PCR using primers specific for the *CYP27B1* gene. The data represent analyses of two independent clones with triplicate analyses of each clone. Each experimental control was set up as 1 and each experimental treatment was normalized to its control.

The results regarding the differential growth inhibitory response of 1,25D_3_ on cell proliferation in the VDRff and VDRFF cell lines prompted the investigation of whether *CYP24A1*, a direct Vitamin D target gene and catabolizing enzyme [1], was also differentially regulated in these cell lines. The expression levels of *CYP24A1* mRNA and protein were compared in the three cell lines after 1,25D_3_ treatment. As demonstrated in Figure 3A, *CYP24A1* mRNA was induced at a 1.8 fold higher rate in VDRFF expressing cells than in VDRff cells 24 hours post 1,25D_3_ treatment (P<0.05). Similarly, *CYP24* mRNA levels in VDRFF cells were 4 fold higher compared to Vector controls (P<0.01). Comparable results were observed in parental MCF-7 cells transiently overexpressing the two VDR variants at similar VDR levels (data not shown). Consistent with the mRNA expression, CYP24 protein levels were also significantly upregulated in VDRFF cells compared to the other two cell lines (Figure 3B). To further establish the effect of VDRFF on *CYP24* transactivation, effects of vitamin D on the *CYP24* promoter activity were evaluated by luciferase assay. As expected, *CYP24* reporter activity was significantly higher in MCF-7-VDRFF cells compared to VDRff overexpressing cells after incubation with 1,25D_3_ (P<0.001), (data not shown). To confirm these findings, we conducted an experiment with MDA-MB231 human breast cancer cells transiently overexpressing the two different VDR variants under identical conditions and found similar results (VDRff treatment vs VDRFF treatment, P<0.01; Figure 3C). These results suggest that although the VDR in Vector control and VDRff cells is functional, VDRFF is more potent in mediating 1,25D_3_ upregulation of CYP24.

*CYP27B1* is important in the synthesis of the active form of Vitamin D from its precursor 25(OH)D_3_ [18] and has been found to be expressed in MCF-7 cells both at the mRNA and protein levels [19]. Therefore, the effect of *FokI* polymorphism on *CYP27B1* mRNA expression was explored in MCF-7 cells. As shown in Figure 3D, the *FokI* polymorphism did not alter *CYP27B1* mRNA expression after 1,25D_3_ treatment.

### VDRFF is an effective suppressor of estrogen receptor mediated signaling

**Figure 4:**
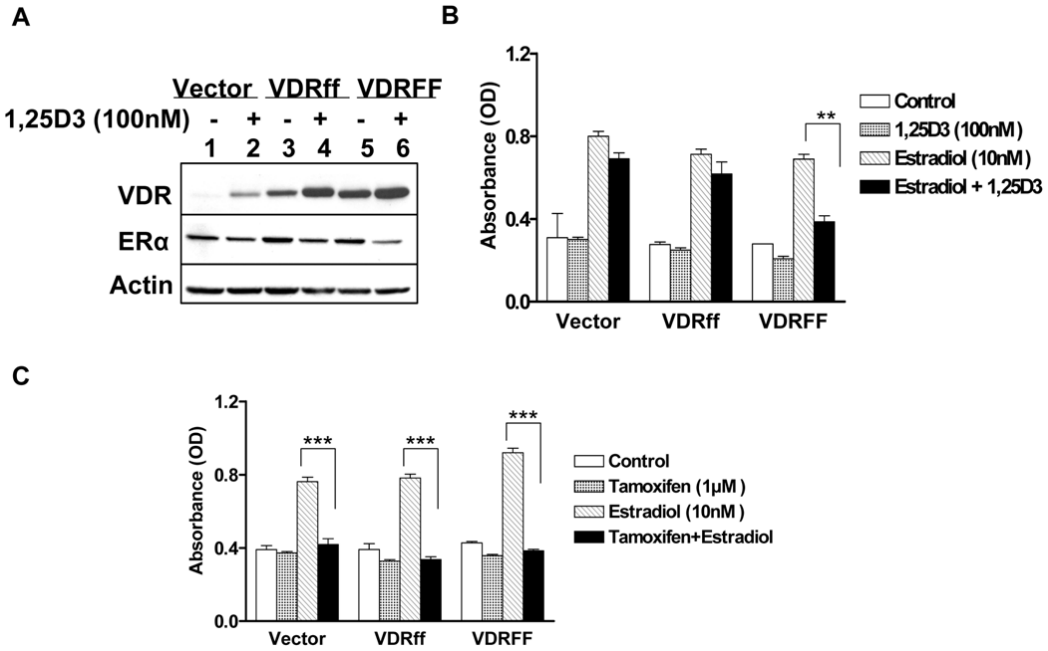
Effects of 1,25D_3_ on estrogen receptor mediated signaling in relation to selective VDR variants. (A) The cells were treated with 100 nM of 1,25D_3_ for 48 h and the expressions of VDR and ERα proteins were determined. (B) The same cells were incubated with (E2) in the presence or absence of 100 nM 1,25D_3_ for 4 days keeping appropriate controls and subjected to the crystal violet assay. (C) The indicated cells were treated with E2 (10nM) in the presence or absence of 1 µM tamoxifen for 4 days and subjected to the crystal violet assay. The data represent analyses of two independent clones with triplicate analyses of each clone. Bars, mean ±SD; **P<0.01, ***P<0.001 (one-way ANOVA test).

It is well documented that ER positive breast cancer growth is dependent on estrogen and that 1,25D_3_ down-regulates ERα expression in MCF-7 cells [20]. To uncover the role of VDRff and VDRFF on ERα signaling, the cells were exposed to 1,25D_3_ for forty eight hours and ERα protein expression was assessed. As demonstrated in Figure 4A ERα protein expression was substantially downregulated by 62% in VDRFF cells compared to 20% in Vector and VDRff cells after 1,25D_3_ treatment. The protein band intensities were calculated after actin normalization utilizing the UnScan-It gel program (Silk Scientific, Inc.). ERα expression was consistently downregulated in response to 1,25D_3_ in parental MCF-7 cells overexpressing increasing concentrations of VDRFF plasmid in contrast to increasing concentrations of VDRff plasmid at equal VDR levels (data not shown), indicating that VDRFF is more effective in mediating vitamin D action. To further identify the effects of VDR *FokI* polymorphism on estrogen mediated signaling, the cells were treated with estradiol in the presence or absence of 1,25D_3_. As shown in Figure 4B, estrogen induced cell growth was significantly inhibited by 1,25D_3_ in VDRFF overexpressing cells (P<0. 01) while no significant inhibition was observed in Vector and VDRff cells 4 days after treatments. Similar results were obtained 7 days after treatments (data not shown). Cumulatively, these results provide support for defining the VDRff and VDRFF variants as differential mediators of vitamin D action with the VDRFF form as the more active modulator. Next, the effect of the anti-estrogen tamoxifen was evaluated on estrogen stimulated cell proliferation as it is well known to negatively arbitrate this pathway. It is well established that tamoxifen inhibits estrogen mediated signaling by binding to ERα and thereby preventing the activation of estrogen responsive genes. Thus, Vector, VDRff and VDRFF cells were treated with estradiol in the presence or absence of tamoxifen for 4 days. As shown in Figure 4C, tamoxifen equally inhibited estrogen induced cell growth in MCF-7-Vector, MCF-7-VDRff and MCF-7-VDRFF cell lines (P<0.001), whereas tamoxifen as expected, had no effect in the absence of estradiol. These results indicate that cells with the *FokI* polymorphism are differentially responsive only to vitamin D and not anti-estrogens.

### VDRFF protein is more stable than VDRff protein

**Figure 5:**
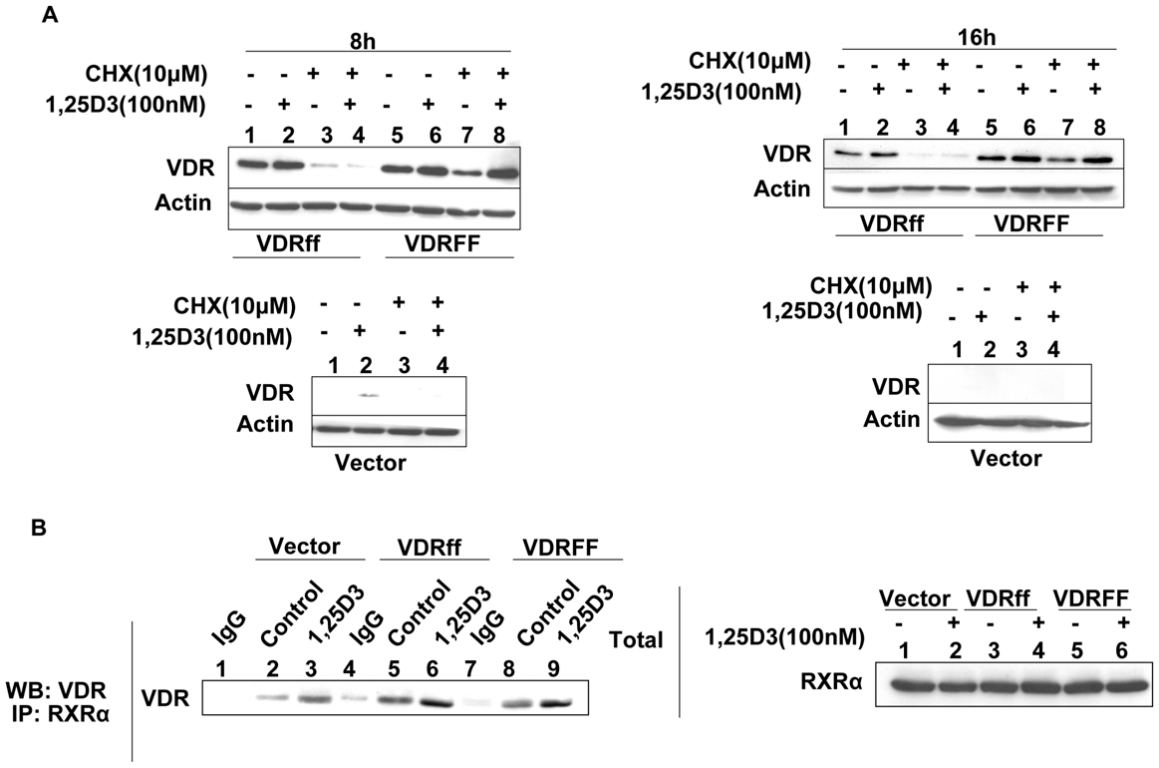
Effects of 1,25D_3_ on the stability of VDR protein in MCF-7-VDR *FokI* polymorphic cells. (A) The cells were treated with 10 µM cycloheximide in the presence or absence of 1,25D_3_ as described in Methods and the protein lysates were processed for VDR using western blot analysis. (B) The indicated cells were treated with 100 nM of 1,25D_3_ for 24 h and total lysates were subjected to co-immunoprecipitation using an RXRα rabbit polyclonal antibody; immunoprecipitates (left panel). The total lysates were evaluated for RXRα expression (right panel).

It is well established that 1,25D_3_ stabilizes VDR protein [21]. Therefore, to determine whether the difference between the two VDR variants was due to disparities in VDR protein stability, the cells were exposed to the protein synthesis inhibitor cycloheximide (CHX, 10 µM) in the presence or absence of 1,25D_3_ for eight and sixteen hours. As shown in Figure 5A (lanes 3 and 4), CHX treatment inhibited the synthesis of basal VDR protein levels as well as 1,25D_3_ induced VDR protein levels in VDRff cells. In VDRFF cells however, basal VDR expression was slightly reduced compared to control but 1,25D_3_ treatment rendered the receptor resistant to CHX effects (Figure 5A, lanes 7 and 8). These observations suggest that the VDRFF protein is more stable and that 1,25D_3_ increases the half life of VDR in VDRFF cells more effectively.

### VDR *FokI* polymorphism has no effect on VDR and RXRα heterodimerization

Jurutka et al identified VDRFF as possessing a stronger affinity to bind Transcription Factor IIB, indicating one possible mechanism for this particular variant's enhanced transcriptional activity [22]. To identify alternative mechanisms for the apparent disparities in the *FokI* polymorphism's sensitivity to 1,25D_3_, we compared the ability of *FokI* variants to heterodimerize with RXRα, an established partner of VDR. Therefore, Vector, VDRff and VDRFF cells were treated with 1,25D_3_ for twenty four hours and subsequently subjected to co-immunoprecipitation using a rabbit polyclonal antibody against RXRα. The immunoprecipitates were subjected to western blot analysis using antibodies directed against VDR protein (Figure 5B, left panel). Total lysates were also analyzed for RXRα protein as a loading control (Figure 5B right panel). As depicted in Figure 5B (left panel), the two receptors bound together and this binding was enhanced with 1,25D_3_ treatment in Vector, VDRff and VDRFF cells. However, no difference was observed in the dimerization of the VDR two variants with RXRα. Thus, this result suggests that the differential effects observed in these cell lines are not due to enhanced RXRα and VDR association.

### Pro-inflammatory genes and anti-apoptotic genes are upregulated in VDRff cells

**Figure 6:**
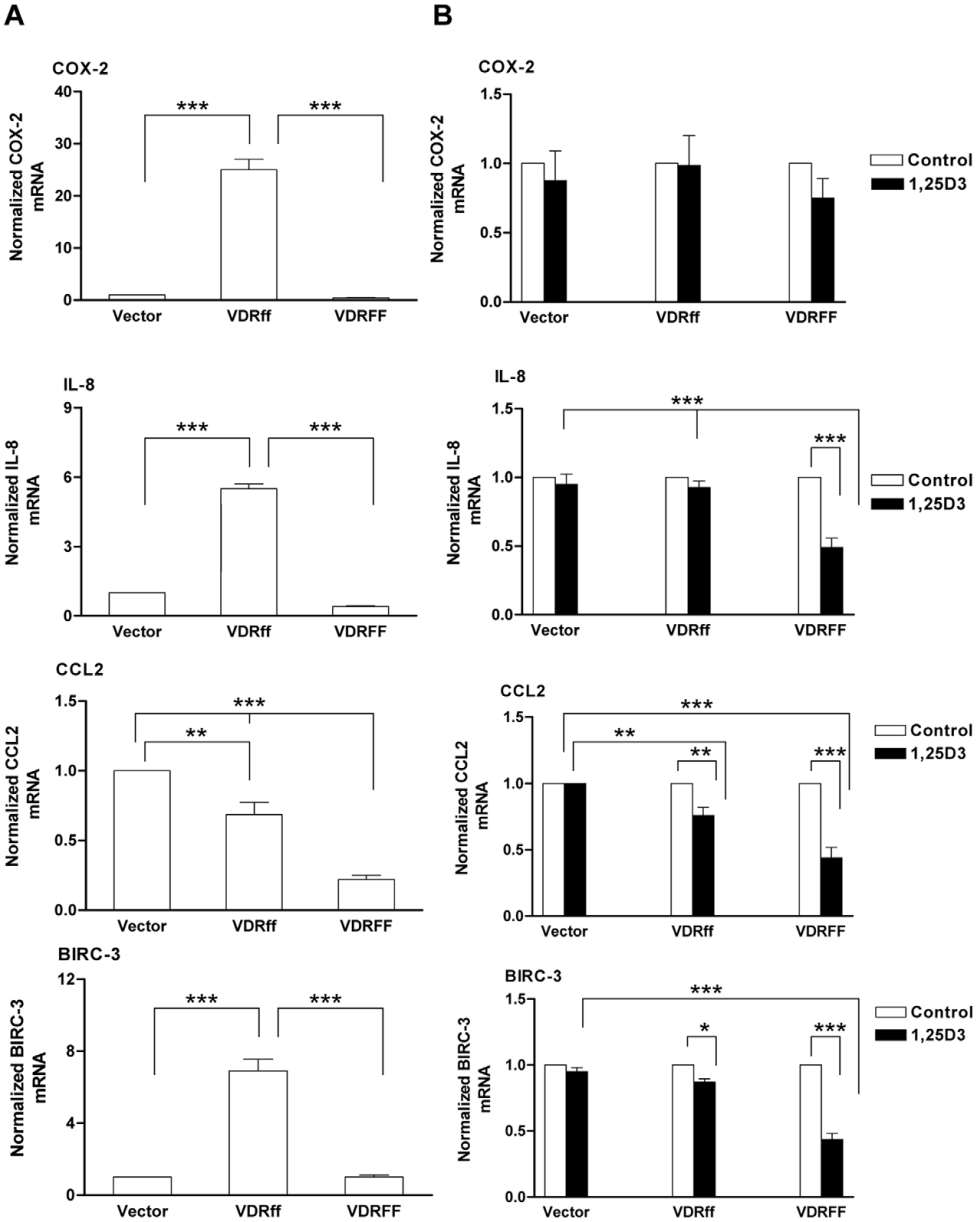
Comparative expression of pro-inflammatory genes in VDRff and VDRFF cells. (A) Total RNA was isolated from the cells and cDNA was subjected to qRT-PCR using primers specific to the indicated genes. The data represent analyses of three independent experiments. Bars, mean ±SD; **P<0.01, ***P<0.001 (one-way ANOVA test). (B) The cells were treated with 100 nM of 1,25D_3_ for 24 h and total RNA was evaluated for the expression of selected genes using qRT-PCR. The data represent analyses of three independent experiments. Bars, mean ±SD; *P<0.05, **P<0.01, ***P<0.001(one-way ANOVA test).

The results presented thus far characterized VDRff and VDRFF as distinct, diversely modulating 1,25D_3_ action. Therefore, a Signal Transduction Pathway Finder PCR Array (SA, Biosciences) was employed to ascertain whether these VDR alleles differentially regulate any genes in the signal transduction pathway independent of 1,25D_3_ treatment. Table 1 shows various differentially expressed genes with fold changes of 2.5 or higher in VDRff cells compared to their VDRFF counterpart. Notably, the pro-inflammatory genes *Cyclooxygenase-2* (*COX-2/PTGS2*) [23], *Interleukin-8* (*IL-8*) [24] and *Chemokine C-C Motif Ligand 2* (*CCL2/MCP-1*) [25] were upregulated 14, 52, and 5 fold respectively in VDRff cells compared to VDRFF cells. Additionally, the apoptosis suppressor *Baculoviral IAP repeat containing 3* (*BIRC-3/cIAP2*) [26] was upregulated 8 fold. The differential expression of these genes in *FF* and *ff* variants was confirmed by qRT-PCR. As illustrated in Figure 6A, basal upregulation of several pro-inflammatory genes in VDRff expressing cells was observed compared to VDRFF. These genes include *COX-2* (P<0.001), *IL-8* (P<0.001), *CCL2* (P<0.001) and *BIRC-3* (P<0.001). The effect of 1,25D_3_ on the *COX-2, IL-8, CCL2* and *BIRC-3* genes was also assessed in the three cell lines. As illustrated in Figure 6B, 1,25D_3_ treatment of VDRFF cells significantly downregulated the expression of *IL-8* (P<0.001, VDRFF control vs. treatment), *CCL2* (P<0.001, VDRFF control vs. treatment*) and BIRC-3*(P<0.001, VDRFF control vs. treatment). In VDRff cells, only *CCL2* (P<0.01) and *BIRC-3*(P<0.05) were downregulated in response to 1,25D_3_ treatment. Unexpectedly, however, *COX-2* mRNA was not significantly downregulated in VDRFF cells in response to 1,25D_3_.

**Table 1:** A comparison of differentially regulated genes in VDRff and VDRFF cells.

Gene ID	Gene Symbol	Gene Name	Fold Regulation (*VDRff/VDRFF*)	Biological Function
NM_000963	*PTGS2 (COX-2)*	*Prostaglandin-endoperoxide synthase 2*	+14.12	Pro-inflammatory, breast cancer metastasis[23]
NM_000584	*IL8*	*Interleukin 8*	+52.71	Pro-inflammatory, breast cancer metastasis [24]
NM_002982	*CCL2*	*Chemokine (C-C motif) ligand 2*	+5.35	Pro-inflammatory, breast cancer metastasis [25]
NM_001165	*BIRC3(cIAP)*	*Baculoviral IAP repeat containing 3*	+8.69	Apoptosis suppressor [26]
NM_005522	*HOXA1*	*Homobox A1*	+5.35	Sequence specific transcription factor, apoptosis inhibitor in breast cancer [34]
NM_000594	*TNF*	*Tumor necrosis factor(TNF superfamily member 2)*	−5.21	Cytokine, induces cell death or under certain conditions, induces cell proliferation and differentiation [35]-[36]
NM_000586	*IL2*	*Interleukin 2*	−3.94	Produced by T cells, crucial for the regulation of the immune response [37]
NM_014207	*CD5*	*CD5 molecule*	−2.79	Repressor of T-cell and B-cell receptor signaling [38]
NM_003998	*NFKB1*	*Nuclear factor of kappa light*	+2.5	Transcription factor, pro-inflammatory [39]

## Discussion

This study is the first report providing evidence for distinct functional differences between VDRff and VDRFF *FokI* genetic variants in MCF-7 breast cancer cells. Here we observed that although VDRff and VDRFF overexpressing cells are morphologically similar, the VDRFF variant is more efficient in mediating 1,25D_3_ action. We previously reported that 1α (OH)D_5_, a less calcemic analog of 1,25D_3_, inhibited the proliferation of BT474 and ZR-75-1 breast cancer cells by 50% and 30% respectively after 72 hours of treatment [27]. Interestingly, BT474 cells are homozygous for the FF allele while ZR-75-1 cells are homozygous for the ff allele [8]. Thus, such disparities in sensitivity to vitamin D may be attributed to polymorphisms in the VDR gene. Consequently, it is highly plausible that a more effective transcription factor such as VDRFF positively influences vitamin D action *in vitro* and *in vivo*.

The regulation of *CYP24* by vitamin D is well characterized. Previously it has been reported that *CYP24A1* mRNA was upregulated 7000 fold in the presence of 1,25D_3_ in several melanoma cell lines, concomitant with significant growth inhibition [28]. In contrast, *CYP24A1* mRNA induction was 100 times less in other melanoma cells impervious to 1,25D_3_ antiproliferative effects [28]. Consistent with this finding, we observed, in response to 1,25D_3_, that CYP24 mRNA and protein were significantly induced at a higher rate in MCF-7-VDRFF cells compared to MCF-7-VDRff cells, further strengthening the conclusion that the VDRFF variant instigates a more intense response to vitamin D than its VDRff counterpart. Interestingly, despite the effects of VDRFF on *CYP24A1*, no regulation was observed in *CYP27B1* mRNA in any of the three cell lines after exposure to 1,25D_3_. In line with this, it has been reported that 1,25D_3_ has no effect on *CYP27B1* activity in parental MCF-7 cells possibly as a result of selective promoter usage [18].

Previous studies have shown that vitamin D analogs and parent agents suppress the cell proliferation of ER+ cells more effectively compared to ER- cells. Vitamin D analogs do not provide any significant cell inhibitory activity in MDA-MB-231 cells, whereas these vitamin D analogs are antiproliferative in ER+ cells [16]. The results presented in this report indicate that VDRFF may facilitate the antiproliferative effects on estrogen mediated cell growth in part by down-regulating ERα expression as it was significantly reduced in these cells compared to the other two cell lines after 1,25D_3_ treatment. Together these observations demonstrate that the VDRFF variant is an effective negative modulator of 1,25D_3_ on estrogen receptor mediated signaling and that breast cancer patients whose cells express the *FF* genotype may benefit from vitamin D therapy.

Another possible mechanism by which VDRFF enhances vitamin D efficacy may be through increased receptor protein stability. Our results revealed that 1,25D_3_ treatment stabilized both VDRff and VDRFF proteins. However, VDRFF cells were resistant to the effects of the protein synthesis inhibitor cycloheximide even without 1,25D_3_ treatment, indicating that the VDRFF protein may be more stable than VDRff protein. It has been reported that the N terminal sequence of a protein is often determinant of its stability [29], thus, it is possible that the differential stability of VDRff and VDRFF may be due to a difference in their N terminal sequence [22]. Collectively, our results suggest that both protein stability and higher activity of the VDRFF variant contribute to this variant's enhanced response to vitamin D in breast cancer cells.

One major change observed was that the basal expression of pro-inflammatory genes *COX-2*, *IL-8*, *CCL2* and *BIRC-3* was significantly upregulated in cells constitutively overexpressing the VDRff variant. Notably, the expression of *IL-8*, *CCL2* and *BIRC-3* was downregulated by 1,25D_3_ treatment significantly in VDRFF cells compared to Vector control and VDRff cells. Recent accumulating evidence describes *COX-2* as a candidate breast cancer metastases gene [23], [30]–[32]. For example, it has been shown that *COX-2* is one of the genes involved in potentiating breast cancer metastasis to the brain and lung respectively [31], [32]. Notably, elevated *COX-2* and *IL-8* expression in breast cancer patients has been positively linked with an unfavorable prognosis and accelerated progression to metastatic disease [30]. *COX-2* overexpression in MDA-MB231 breast cancer cells and MCF10A breast epithelial cells has also been correlated with increased *IL-8* expression and *COX-2* antagonists such as NS-398 have been shown to down-regulate *IL-8* [30]. Therefore, it can be inferred that *COX-2* and *IL-8* may cooperate in promoting the invasion of breast cancer cells with a VDRff genotype to other organs. Similarly, consistent with our results, *CCL2* has also recently been reported to instigate breast cancer metastasis to the lung and bone [25]. Together, these observations suggest that increased expression of pro-inflammatory genes such as *COX2*, *IL-8* and *CCL2* may characterize the VDRff variant in breast cancer cells as a possible clinical marker for aggressive tumors. It is important to note that VDRff itself does not cause the aggressive phenotype, but due to its increased transcriptional activity of genes implicated in an aggressive phenotype, the VDRff genotype may fail to protect normal cells from oncogenic insults over time.

As described in the Results, we observed upregulation of *BIRC-3* mRNA in VDRff cells indicating that breast cancer cells expressing this genotype may be resistant to apoptosis, potentially contributing to an unfavorable prognosis. In support of this, knockdown of *XIAP*, a related member of the *BIRC-3* anti-apoptotic family in MCF-7 breast cancer cells sensitized these cells to apoptosis mediated by chemotherapeutic drugs [33]. The disparate expression of pro-metastasis and anti-apoptotic genes in these cell lines may be due to the differential regulation of the promoters of these genes by VDRff and VDRFF.

Although numerous epidemiological investigations on VDR *FokI* polymorphism have painted a contradictory picture, a recent meta-analysis of twenty one case-control studies significantly correlating the VDRff variant with an overall enhanced breast cancer risk [13], substantiates the experimental findings presented in this report. Thus, a VDRff genotype may be classified as one of numerous determinants underlying a genetic susceptibility to a virulent form of breast cancer, whereas cells expressing VDRFF may be better suited for vitamin D treatment. Therefore, these observations provide an additional genetic marker that may be clinically useful in deciphering an individual's response to vitamin D treatments.

## References

[1] Deeb KK, Trump DL, Johnson CS (2007). Vitamin D Signaling pathways in cancer: potential for anticancer therapeutics.. Nat Rev Cancer.

[2] Crew KD, Shane E, Cremers S, McMahon DJ, Irani D (2009). High prevalence of Vitamin D deficiency despite supplementation in premenoupausal women with breast cancer undergoing adjuvant chemotherapy.. J Clin Oncol.

[3] Goodwin PJ, Ennis M, Pritchard KI, Koo J, Hood N (2009). Prognostic effects of 25-hydroxyvitamin D levels in early breast cancer.. J Clin Oncol.

[4] Ellison TI, Eckert RL, MacDonald PN (2007). Evidence for 1,25-dihydroxyvitamin D3-independent transactivation by the vitamin D receptor: uncoupling the receptor and ligand in keratinocytes.. J Biol Chem.

[5] Zinser GM, McEleney K, Welsh J (2003). Characterization of mammary tumor cell lines from wild type and vitamin D3 receptor knockout mice.. Mol Cell Endocrinol.

[6] Whitfield GK, Remus LS, Jurutka PW, Zitzer H, Oza AK (2001). Functionally relevant polymorphisms in the human nuclear vitamin D receptor gene.. Mol Cell Endocrinol.

[7] Trabert B, Malone KE, Daling JR, Doody DR, Bernstein L (2007). Vitamin D receptor polymorphisms and breast cancer risk in a large population-based case-control study of Caucasian and African-American women.. Breast Cancer Res.

[8] Guy M, Lowe LC, Bretherton-Watt D, Mansi JL, Peckitt C (2004). Vitamin D receptor gene polymorphisms and breast cancer risk.. Clin Cancer Res.

[9] Sinotte M, Rousseau F, Ayotte P, Dewailly E, Diorio C (2008). Vitamin D receptor polymorphisms (FokI, BsmI) and breast cancer risk: association replication in two case-control studies within French Canadian population.. Endocr Relat Cancer.

[10] Chen WY, Berone-Johnson ER, Hunter DJ, Willett WC, Hankinson SE (2005). Association between polymorphism in the vitamin D receptor and breast cancer risk.. Cancer Epidemiol Biomarkers Prev.

[11] McCullough ML, Stevens VL, Diver WR, Feigelson HS, Rodriguez C (2007). Vitamin D pathway gene polymorphisms, diet, and risk of postmenopausal breast cancer: a nested case-control study.. Breast Cancer Res.

[12] Raimondi S, Johansson H, Maisonneuve P, Gandini S (2009). Review and meta-analysis on vitamin D receptor polymorphisms and cancer risk.. Carcinogenesis.

[13] Tang C, Chen N, Wu M, Yuan H, Du Y (2009). FokI polymorphism of vitamin D receptor gene contributes to breast cancer susceptibility: a meta-analysis.. Breast Cancer Res Treat.

[14] Alimirah F, Chen J, Xin H, Choubey D (2006). Androgen receptor autoregulates its expression by a negative feedback loop through upregulation of IFI16 protein.. FEBS Lett.

[15] Whyte L, Huang YY, Torres K, Mehta RG (2007). Molecular mechanisms of resveratrol action in lung cancer cells using dual protein and microarray analyses.. Cancer Res.

[16] Peng X, Jhaveri P, Hussain-Hakimjee EA, Mehta RG (2007). Overexpression of ER and VDR is not sufficient to make ER-negative MDA-MB231 breast cancer cells responsive to 1α-hydroxyvitamin D5.. Carcinogenesis.

[17] Jensen SS, Madsen MW, Lukas J, Bartek J, Binderup L (2002). Sensitivity to growth suppression by 1alpha,25-dihydroxyvitamin D(3) among MCF-7 clones correlates with Vitamin D receptor protein induction.. J Steroid Biochem Mol Biol.

[18] Turunen MM, Dunlop TW, Carlberg C, Väisänen S (2007). Selective use of multiple vitamin D response elements underlies the 1 alpha,25-dihydroxyvitamin D3-mediated negative regulation of the human CYP27B1 gene.. Nucleic Acids Res.

[19] Kemmis CM, Salvador SM, Smith KM, Welsh JE (2006). Human mammary epithelial cells express CYP27B1 and are growth inhibited by 25-hydroxyvitamin D-3, the major circulating form of vitamin D-3.. J Nutr.

[20] Swami S, Krishnan AV, Feldman D (2000). 1alpha,25-Dihydroxyvitamin D3 down-regulates estrogen receptor abundance and suppresses estrogen actions in MCF-7 human breast cancer cells.. Clin Cancer Res.

[21] Li XY, Boudjelal M, Xiao JH, Peng ZH, Asuru A (1999). 1,25-Dihydroxyvitamin D3 increases nuclear vitamin D3 receptors by blocking ubiquitin/proteasome-mediated degradation in human skin.. Mol Endocrinol.

[22] Jurutka PW, Remus LS, Whitfield GK, Thompson PD, Hsieh JC (2000). The polymorphic N terminus in human vitamin D receptor isoforms influences transcriptional activity by modulating interaction with transcription factor IIB.. Mol Endocrinol.

[23] Pan MR, Hou MF, Chang HC, Hung WE (2008). Cyclooxygenase-2 up-regulates CCR7 via EP2/EP4 receptor signaling pathways to enhance lymphatic invasion of breast cancer.. J Biol Chem.

[24] De Larco JE, Wuertz BR, Rosner KA, Erickson SA, Gamache DE (2001). A potential role for Interleukin-8 in the metastatic phenotype of breast carcinoma cells.. Am J Pathol.

[25] Lu X, Kang Y (2009). Chemokine (C-C motif) ligand 2 engages CCR2+ stromal cells of monocytic origin to promote breast cancer metastasis to lung and bone.. J Biol Chem.

[26] Varfolomeev E, Vucic D (2008). (Un)expected roles of c-IAPs in apoptotic and NFkappaB signaling pathways.. Cell Cycle.

[27] Hussain EA, Mehta RR, Ray R, Das Gupta TK, Mehta RG (2003). Efficacy and mechanism of action of 1α-hydroxy-24-ethyl-cholecalciferol (1α[OH]D5 in breast cancer prevention and therapy.. Recent Results Cancer Res.

[28] Reichrath J, Rech M, Moeini M, Meese E, Tilgen W (2007). In vitro comparison of the vitamin D endocrine system in 1,25(OH)2D3 responsive and resistant melanoma cells.. Cancer Biol Ther.

[29] Varshavsky A (1997). The N-end rule pathway of protein degradation.. Genes Cells.

[30] Singh B, Berry JA, Vincent LE, Lucci A (2006). Involvement of IL-8 in COX-2-mediated bone metastases from breast cancer. J Surg Res.

[31] Bos PD, Zhang XH, Nadal C, Shu W, Gomis RR (2009). Genes that mediate breast cancer metastasis to the brain.. Nature.

[32] Minn AJ, Gupta GP, Siegel PM, Bos PD, Shu W (2005). Genes that mediate breast cancer metastasis to lung.. Nature.

[33] Lima RT, Martins LM, Guimaraes JE, Sambade C, Vasconcelos MH (2004). Specific downregulation of bcl-2 and xIAP by RNAi enhances the effects of chemotherapeutic agents in MCF-7 human breast cancer cells.. Cancer Gene Ther.

[34] Zhang X, Zhu T, Chen Y, Hichem MC, Lee K (2003). Human growth hormone-regulated HOXA1 is a human mammary epithelial oncogene.. J Biol Chem.

[35] Warren MA, Shoemaker SF, Shealy DJ, Bshar W, Ip MM (2009). Tumor necrosis factor deficiency inhibits mammary tumorigenesis and a tumor necrosis factor neutralizing antibody decreases mammary tumor growth in neu/erbB2 transgenic mice.. Mol Cancer Ther.

[36] Kang JX, Liu J, Wang J, He C, Li FP (2005). The extract of huanglian, a medicinal herb, induces cell growth arrest and apoptosis by upregulation of interferon-beta and TNF-alpha in human breast cancer cells.. Carcinogenesis.

[37] Waldmann TA (2006). The biology of interleukin-2 and interleukin-15: implications for cancer therapy and vaccine design.. Nat Rev Immunol.

[38] Dalloul A (2009). CD5: a safeguard against autoimmunity and a shield for cancer cells.. Autoimmun Rev.

[39] Yamaguchi N, Ito T, Azuma S, Ito E, Honma R (2009). Constitutive activation of nuclear factor-kappaB is preferentially involved in the proliferation of basal-like subtype breast cancer cell lines.. Cancer Sci.

